# A Duration-Dependent Interaction Between High-Intensity Light and Unrestricted Vision in the Drive for Myopia Control

**DOI:** 10.1167/iovs.64.3.31

**Published:** 2023-03-23

**Authors:** Sayantan Biswas, Arumugam R. Muralidharan, Bjorn Kaijun Betzler, Joanna Marie Fianza Busoy, Veluchamy A. Barathi, Royston K. Y. Tan, Wan Yu Shermaine Low, Dan Milea, Biten K. Kathrani, Noel A. Brennan, Raymond P. Najjar

**Affiliations:** 1 Singapore Eye Research Institute, Singapore; 2 Ophthalmology and Visual Science Academic Clinical Program, Duke-NUS Medical School, Singapore; 3 Yong Loo Lin School of Medicine, National University of Singapore, Singapore; 4 Singapore National Eye Centre, Singapore; 5 Johnson and Johnson Vision Care, Jacksonville, Florida, United States; 6 Eye N’ Brain Research Group, Department of Ophthalmology, Yong Loo Lin School of Medicine, National University of Singapore, Singapore; 7 Center for Innovation & Precision Eye Health, Yong Loo Lin School of Medicine, National University of Singapore, Singapore

**Keywords:** myopia, animal model, unrestricted vision, optical defocus, high-intensity light, axial length, choroid, outdoor time

## Abstract

**Purpose:**

To evaluate the duration-dependent and synergetic impact of high-intensity light (HL) and unrestricted vision (UnV) on lens-induced myopia (LIM) development in chickens.

**Methods:**

Myopia was induced in one eye in chicks (10 groups, *n* = 126) from day 1 posthatching (D1) until day 8 (D8) using –10 diopter (D) lenses. Fellow eyes remained uncovered as controls. Nine groups were exposed daily to 2, 4, or 6 hours of HL (15,000 lux), UnV (removal of –10 D lens), or both (HL + UnV). One group served as the LIM group without any interventions. Ocular axial length (AL), refractive error, and choroidal thickness were measured on D1, D4, and D8. Outcome measures are expressed as interocular difference (IOD = experimental eye – control eye) ± SEM.

**Results:**

By D8, LIM increased AL (0.36 ± 0.04 mm), myopic refraction (−9.02 ± 0.37 D), and choroidal thinning (−90.27 ± 16.44 µm) in the LIM group (all, *P* < 0.001). Compared to the LIM group, exposure to 2, 4, or 6 hours of HL, UnV, or HL + UnV reduced myopic refraction in a duration-dependent manner, with UnV being more effective than HL (*P* < 0.05). Only 6 hours of HL + UnV (not 2 or 4 hours) prevented LIM and was more effective than UnV (*P* = 0.004) or HL (*P* < 0.001) in reducing myopic refraction and more effective than HL (*P* < 0.001) in reducing axial elongation.

**Conclusions:**

Daily exposure to 2, 4, or 6 hours of HL, UnV, or HL + UnV reduced lens-induced myopic refraction in a duration-dependent manner in chickens. Only 6 hours of HL + UnV completely stopped LIM development. The synergetic effect of HL and UnV is dependent on the duration of the interventions.

##  

Myopia, also known as near-sightedness, is one of the most prevalent eye conditions worldwide and has become a public health concern, particularly in East and Southeast Asia.^1^ Axial myopia, the most common form of myopia, involves excessive globe elongation during ocular development, resulting in a mismatch between the axial length (AL) and optical power of the eye.^2^ While the refractive error associated with myopia can be corrected, high myopia is associated with sight-threatening pathologies, including retinal detachment, glaucoma, and myopic maculopathy.^3^

Environmental factors alongside genetics are known to play a significant role in the development of myopia.^4^ Increased outdoor activity in childhood reduces the risk of developing myopia,^5^^–^^7^ independent of physical activity.^8^ This protective impact of outdoor time may be due to a combination of factors, including the intensity and spectral composition of sunlight,^9^^,^^10^ the increased spatial frequency and higher image contrast encountered outdoors,^11^^,^^12^ and the significant differences in the pattern of retinal defocus generated outdoors compared with indoors,^11^^,^^13^ associations that are still under investigation in animal models of myopia.^14^ Because time spent outdoors involves all of these factors simultaneously, the relative protective contribution of each and interaction between them are unknown. Animal models offer an opportunity to explore such associations.

The most commonly used myopia induction methods in animal models are form-deprivation myopia (FDM), in which translucent diffusers are fitted to obscure the animal's vision, and lens-induced myopia (LIM), in which hyperopic defocusing lenses (i.e., negative powered lenses) are fitted to place the focal plane behind the retina and induce axial ocular growth.^15^ While it has been suggested that FDM and LIM have different underlying mechanisms of action,^16^^–^^18^ more evidence is needed to support these claims given the biochemical,^19^^–^^21^ genetic,^22^ and anatomic^23^ similarities between these models of myopia induction, in addition to similarities in their response to pharmacologic agents.^24^

Exposure to high-intensity light (HL; 10,000–25,000 lux per day) can be effective in alleviating FDM in various animal models.^25^^–^^28^ In addition, Ashby et al.^29^ reported that in chicks, the suppression of FDM is even more pronounced when HL was combined with normal vision (diffuser removal) for 15 min/d, compared to experimental groups exposed to either HL (15,000 lux) or diffuser removal alone. The authors postulated that dopamine (DA) release in the chick retina had a graded response to increasing illumination due to diffuser removal, resulting in greater inhibition of axial ocular growth at higher illumination levels.^29^ In studies utilizing LIM, exposure to HL without the defocusing lens removal or unrestricted vision (UnV) significantly slowed LIM development in chicks (15,000 lux for 5 h/d)^30^ but not in monkeys (25,000 lux for 6 h/d),^31^ while exposure to brief periods (1–3 hours) of UnV per day reduced the extent of LIM development in tree shrews,^32^^,^^33^ marmosets,^34^ and chicks.^35^ While LIM and FDM may yield different ocular mechanisms,^16^^,^^17^ lens removal for LIM and normal vision in case of FDM may operate through different pathways. In LIM, UnV may act through a change in optical and visual-spatial information, whereas normal vision in FDM additionally involves neuromodulations associated with increased retinal illumination.^36^^–^^38^

To our knowledge, the dose-dependent and synergetic effects of HL, UnV, and HL + UnV are yet to be investigated in LIM models. This study investigates the interactive impact of different combinations and durations of HL and UnV on ocular axial elongation, refractive error development, choroidal thickness, and other ocular parameters in a chicken model of LIM.

## Methods

### Animals and Enclosure

All animals used in this study were treated in accordance with the ARVO Statement for the Use of Animals in Ophthalmic and Vision Research. The study protocol was approved by the Institutional Animal Care and Use Committee (IACUC 2019/SHS/1479) of the Singapore Experimental Medicine Centre (SEMC). The SEMC is accredited by the Association for Assessment and Accreditation of Laboratory Animal Care International.

In total, 126 one-day-old chicks (mixed Golden Comet/White Leghorn strain) were provided by the National Large Animal Research facility and randomly assigned into 10 groups of 11 to 13 animals each. Animals were raised for 9 days in a 75-cm (length) × 55-cm (width) × 43-cm (height) custom-built enclosure designed to hold two high-intensity light-emitting diode (LED) light fixtures. The enclosure walls were fitted with a square wave grating of a repeated sequence of light and dark bars as accommodative cues. The spatial frequency of the stripes ranged between 0.01 and 0.42 cycles/degree depending on the location of the animal within the enclosure. The same visual environment was maintained across experimental groups to avoid differences in accommodative responses that may affect emmetropization in chicks.^39^ The chicks were fed ad libitum and were raised under a 12/12-hour light-dark cycle from 7 am to 7 pm. The temperature within the enclosure was maintained at 28°C to 32°C. Light and temperature patterns were monitored using a HOBO Pendant data logger (UA-022-64; ONSET, Bourne, MA, USA). At the end of experiment on day 9, chicks were sedated with a mixture of 0.2 mL/kg ketamine and 0.1 mL/kg xylazine and euthanized with an overdose of sodium pentobarbitone to the heart.

### Background and Experimental Light Setup

**Figure 1.**
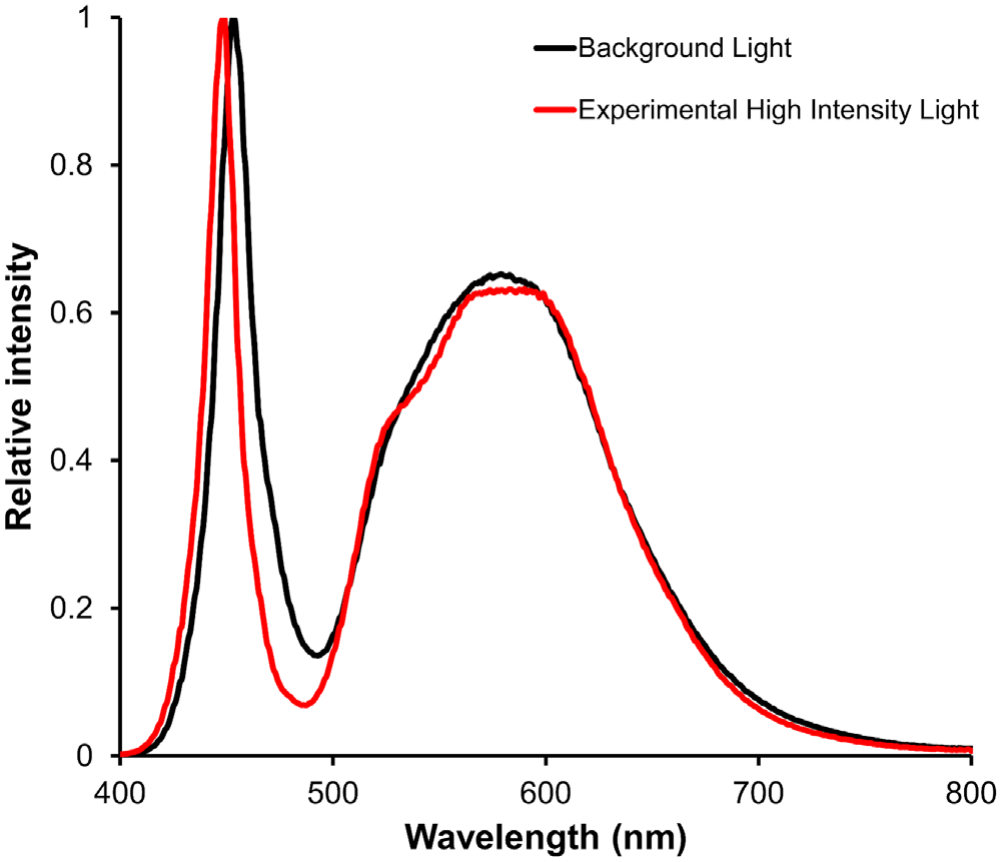
Relative spectral power distribution of the background light (LED, 4000K, 2NFLS-NW LED; Super Bright LED, Inc.) and experimental high-intensity light (LED, 4000K; USHIO Lighting).

All chicks were reared under background lighting (150 lux) during the 12/12-hour light-dark cycle. This was achieved using six strips of ultra-bright LEDs (4000K, 2NFLS-NW LED; Super Bright LED, Inc, St. Louis, MO, USA) fixed over the enclosure. For HL, four LED panels of 64 LEDs each (4000K; USHIO Lighting, Singapore) were used to deliver an average of 15,000 lux when measured in different angles of gazes in the enclosure (up, down, left, right, front, back) (Fig. 1). The light fixtures were controlled using a programmable Helvar DIGIDIM 910 router (Helvar, Dartford Kent, UK). Light levels and spectra were measured using a calibrated radiometer and spectroradiometer (ILT5000 and ILT950; International Light Technologies, Peabody, MA, USA).

### Myopia Induction

Monocular myopia was induced in all chicks from day 1 posthatching (D1) until day 8 (D8) using custom-built concave defocusing lenses (power: −10 ± 0.5 diopters [D], total diameter: 12.5 mm, optic zone diameter: 10 mm, base curve: 6.68 mm; La SER Eye Jewelry, Port St. Lucie, FL, USA). The lens was fitted randomly to one eye of the chick using a custom-built three-dimensional printed lens holder. Considering the diameter of the optic zone (10 mm), an estimated vertex distance (defocusing lens to cornea apex) of 3 mm, and a calculated posterior nodal point to defocusing lens distance of 4.49 mm on D1 in chicks, the open viewing visual angle was estimated to be ∼76.5 degrees. However, these calculations did not take into account changes in pupil size and may have underestimated the open viewing visual angle.^40^ The lens holder can be clipped on or off a separate base piece that was glued to the down surrounding the chick's eye. These lens holders ensured the secure positioning of the lenses on the eyes of the animals and allowed for easy and fast removal during cleaning and light exposure. Lenses were worn for 8 days and were cleaned three times/d to ensure their optical clarity. The fellow eye was kept uncovered and used as a within-animal control.

### Experimental Groups

All 10 groups were subjected to monocular LIM. Nine groups were subjected to different interventions, including either transient HL (15,000 Lux), UnV, or a combination of HL and UnV for one of four durations/d (0, 2, 4, or 6 hours) centered at 12:00 pm. Details on experimental interventions are provided below and in the Table.

**Table.** Details on Experimental Groups and Interventions

					Experimental Interventions	
Experimental Group	Duration of Intervention, h	*N*	Experimental Eye	Control Eye	High-Intensity Light Status (15,000 Lux)	Lens Status
LIM	0	13	−10 D	No lens	Off	Not removed
HL	2	13	−10 D	No lens	On	Not removed
	4	13	−10 D	No lens	On	Not removed
	6	13	−10 D	No lens	On	Not removed
UnV	2	12	−10 D	No lens	Off	Removed
	4	13	−10 D	No lens	Off	Removed
	6	13	−10 D	No lens	Off	Removed
HL + UnV	2	13	−10 D	No lens	On	Removed
	4	12	−10 D	No lens	On	Removed
	6	11	−10 D	No lens	On	Removed

#### LIM Group

Chicks in this group (*n* = 13) were raised under background laboratory light conditions and were not subjected to HL or UnV.

#### High-Intensity Light Groups (LIM + HL)

Three groups (*n* = 13 each) were exposed to 2, 4, or 6 hours of HL (15,000 lux) per day and background light for the remainder of the light cycle. Defocusing lenses were not removed during HL.

#### Unrestricted Vision Groups (LIM + UnV)

Three groups (*n* = 13, 12, and 11) were raised under background light during the light cycle and throughout the experiment. Defocusing lenses were removed for 2, 4, or 6 h/d.

#### High-Intensity Light and Unrestricted Vision Groups (LIM + HL + UnV)

Three groups (*n* = 12, 13, and 13) were exposed to 2, 4, or 6 hours of HL (15,000 lux) per day and background light for the remainder of the light cycle. Defocusing lenses were removed during HL.

### Ocular Measurements In Vivo

On D1, day 4 (D4), and D8, body weight, ocular AL, refractive error, choroidal thickness (CT), central corneal thickness (CCT), and anterior chamber depth (ACD) were measured in the experimental and control eyes of alert, gently handheld chicks. Measurements were taken in a dimly lit room (<5 lux) on all chicks, in a random order, between 12 pm and 5 pm to reduce any impact of circadian rhythm on outcome measures. A lid retractor was used in only a small number of chicks (2 to 3 animals per group, especially on D1) who would not keep their eyelids open. Whenever used, the lid retractor was inserted very carefully by the experimenter so that it does not come into contact with the cornea or obstruct the ophthalmic examination procedure.

### Axial Length

AL was measured using VuMAX HD (Sonomed Escalon, New Hyde Park, NY, USA) A-scan ultrasonography with a probe frequency of 10 MHz as described elsewhere.^41^ AL was measured as the distance between the echo spike corresponding to the anterior surface of the cornea and most anterior spike originating from the retina. Each recorded measurement was the median of 7 to 10 scans.

### Refraction

Ocular refraction was measured using a calibrated automated infrared photo-retinoscope as previously described.^42^ Alert chicks were handheld on an adjustable platform ∼1 m away from the infrared photo-retinoscope. The chick's head was carefully positioned to ensure optimal focus on the chick's eye and first Purkinje image. Pupil size was adjusted for each eye. The median of the most hyperopic refraction readings (i.e., resting refraction) with no accommodative changes was calculated from the continuous refraction trace comprising at least 300 readings over time in each eye.^28^^,^^42^

### Choroidal Thickness and Anterior Segment Characteristics

CT at the posterior pole was measured using posterior segment optical coherence tomography (OCT) (Spectralis; Heidelberg Engineering, Inc., Heidelberg, Germany), and anterior segment (CCT and ACD) was imaged using anterior segment OCT (RTVue; Optovue, Inc., Fremont, CA, USA) as per the protocols described in Najjar et al.^41^ During both procedures, the alert chick's head was gently held and aligned with the OCT camera lens such that the infrared laser beam entered the eye through the center of the pupil. The OCT operator further refined the centration of the pupil, and multiple OCT scans were captured. For posterior segment OCT measurement, the centration was within ±100 µm from the horizontal line. Distance between the inner border of the sclera and the outer border of the retinal pigment epithelium (RPE) was defined as the CT. The average of three thickness measurements of the central cornea was defined as the CCT, whereas ACD was defined as the distance between the central most posterior layer of the cornea and the central most anterior layer of the lens. Measurements were done manually by the first author (SB), who, during measurement sessions, was kept blinded to the eye (LIM or control) and study group (HL, UnV, HL + UnV) conditions.

### Analyses and Statistics

All data are represented as mean ± SEM of the interocular difference (IOD) between the experimental and uncovered control eyes (i.e., experimental [LIM] eye – control eye) to account for interanimal differences in outcome measures, given the mixed breed and large number of animals (*n* = 126 chicks) used in this study. Two-way repeated-measures ANOVA with the factors day, group, and the group × day interaction was used to compare IODs in refraction, AL, CT, ACD, and CCT. A Holm–Sidak method for pairwise multiple comparisons was performed when the omnibus test for interaction effects between group × day was significant. A two-way ANOVA was used to evaluate the interaction between the type of intervention (HL, UnV, HL + UnV) and its duration (0, 2, 4, and 6 hours) on refraction, AL, and CT. A Holm–Sidak method for pairwise multiple comparisons was used when the omnibus test reached statistical significance. The significance level for all statistical tests was set at α = 0.05 with Sidak correction for post hoc pairwise comparisons.

## Results

### Ocular Changes Associated With LIM

**Figure 2.**
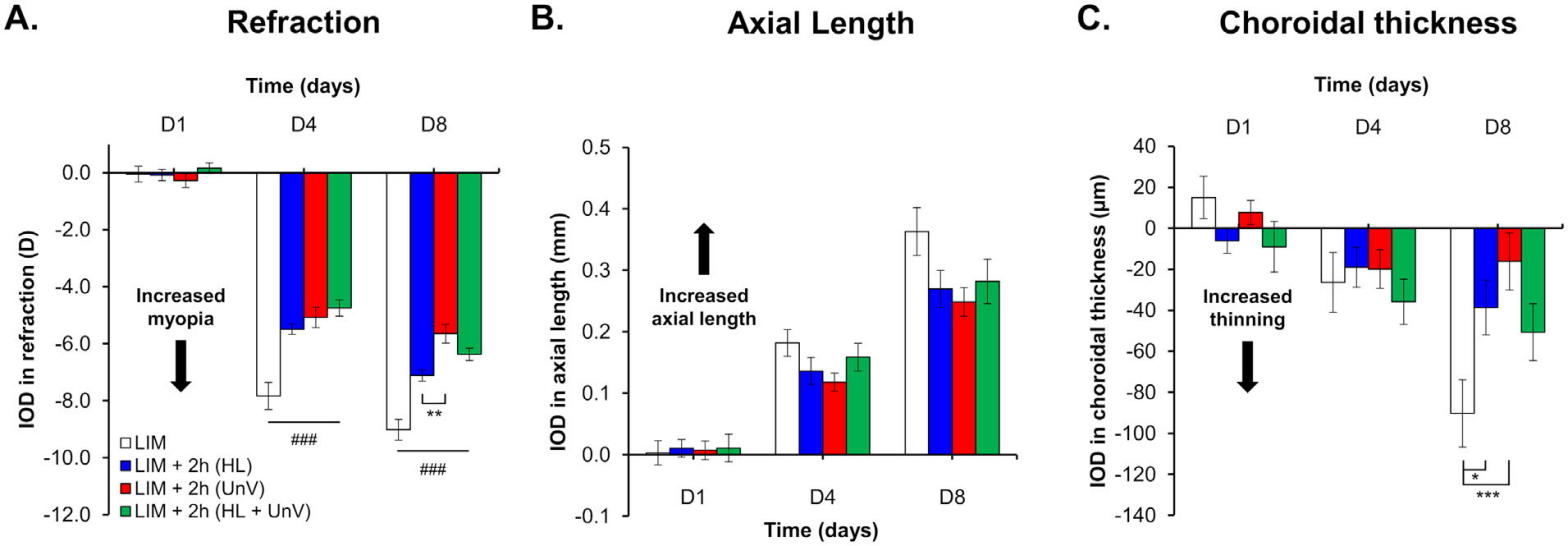
IOD in refraction, axial length, and choroidal thickness on days 1, 4, and 8 of the experimental protocol in the group not exposed to any intervention (LIM) and groups exposed to 2 hours of HL, UnV, or both (HL + UnV). All groups are significantly different from the LIM group: ^###^*P* < 0.001, ^##^*P* < 0.01, ^#^*P* < 0.05. For significant group effect: *P < 0.05, **P < 0.01, ***P < 0.001 (two-way repeated-measures ANOVA).

**Figure 3.**
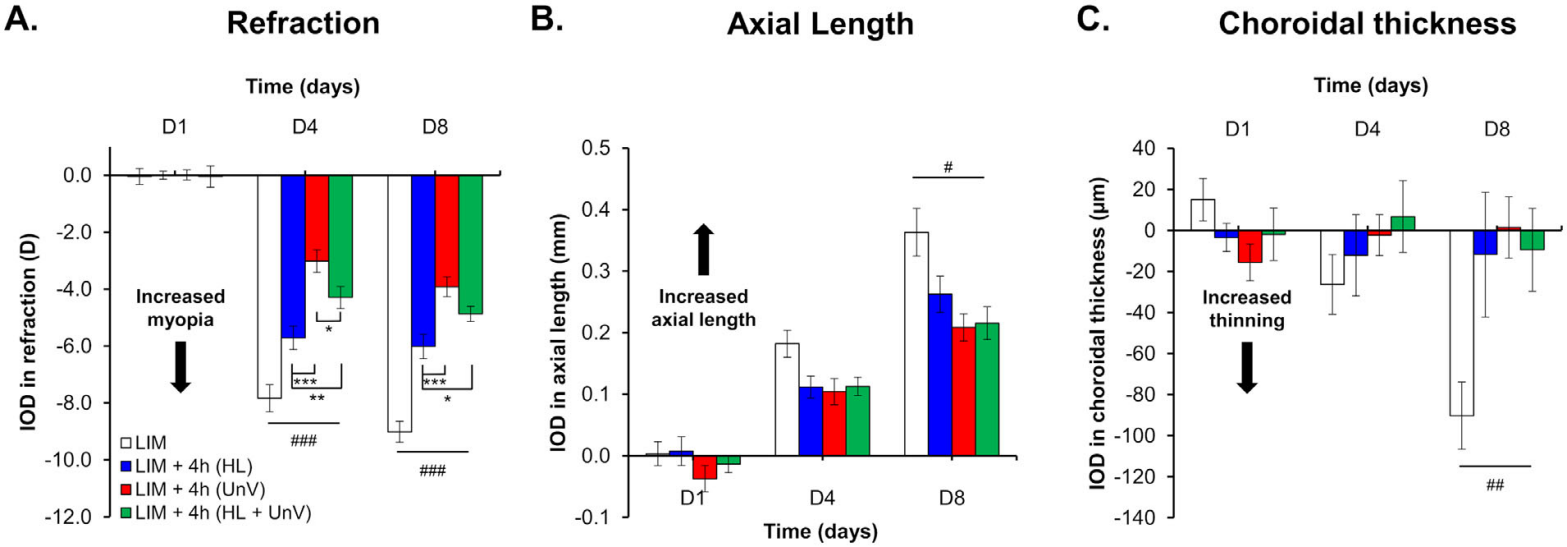
IOD in refraction, axial length, and choroidal thickness on days 1, 4, and 8 of the experimental protocol in the group not exposed to any intervention (LIM) and groups exposed to 4 hours of HL, UnV, or both (HL + UnV). All groups are significantly different from the LIM group: ^###^*P* < 0.001, ^##^*P* < 0.01, ^#^*P* < 0.05. For significant group effect: *P < 0.05, **P < 0.01, ***P < 0.001 (two-way repeated-measures ANOVA).

**Figure 4.**
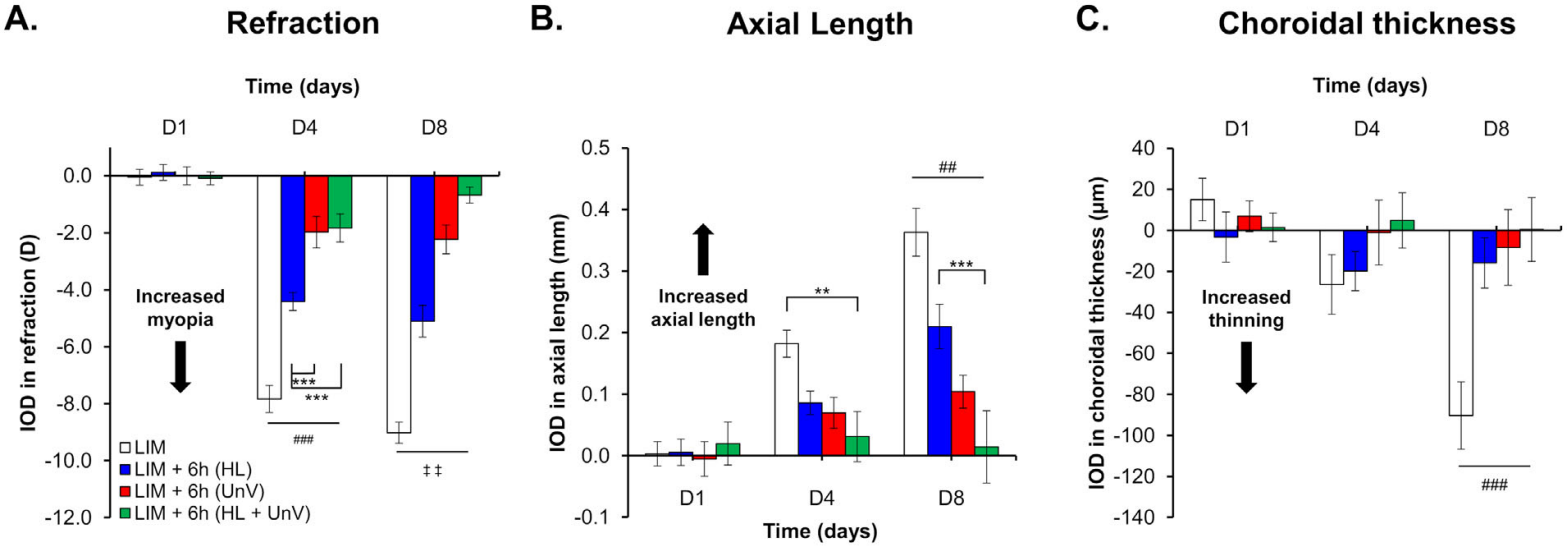
IOD in refraction, axial length, and choroidal thickness on days 1, 4, and 8 of the experimental protocol in the group not exposed to any intervention (LIM) and groups exposed to 6 hours of HL, UnV, or both (HL + UnV). All groups are significantly different from the LIM group: ^###^*P* < 0.001, ^##^*P* < 0.01, ^#^*P* < 0.05. All groups are significantly different from each other: ^‡‡‡^*P* < 0.001, ^‡‡^*P* < 0.01, ^‡^*P* < 0.05. For significant group effect: *P < 0.05, **P < 0.01, ***P < 0.001 (two-way repeated-measures ANOVA).

Compared to the uncovered contralateral control eyes (refraction: +4.83 ± 0.05 D and +5.27 ± 0.06 D by D4 and D8, respectively), the LIM eyes (refraction: −3.00 ± 0.11 D and −3.75 ± 0.07 D by D4 and D8, respectively) displayed a myopic shift in refractive error that predominantly occurred within the first 4 days after −10 D lens wear (IOD: −7.83 ± 0.48 D and −9.02 ± 0.37 D by D4 and D8, respectively). Concurrently, LIM eyes showed an increase in AL (IOD: +0.18 ± 0.02 mm and +0.36 ± 0.04 mm by D4 and D8, respectively) and a decrease in CT (IOD: −26.35 ± 14.55 µm and −90.27 ± 16.44 µm by D4 and D8, respectively) compared to control eyes (all, *P* < 0.001) (Figs. 2, 3, and 4, Supplementary Table S1). The CCT and ACD of LIM eyes were not different from control eyes.

### Impact of 2 Hours of HL, UnV, and HL + UnV

For 2-hour interventions, there was a significant interaction between group and day of intervention for IOD in refraction (*F*(6, 94) = 10.29, *P* < 0.001). By D4 and D8, 2 hours of HL, UnV, and HL + UnV significantly reduced myopic refraction compared to the LIM group (both for D4 and D8, *P* < 0.001). Two hours of UnV, however, was more efficient than 2 hours of HL in reducing myopic refraction on D8 (*P* = 0.001). HL + UnV was as effective as HL and UnV in reducing myopic refraction on D4 and D8 (*P* > 0.05) (Fig. 2A). IOD in AL was only dependent on the day of the intervention (*F*(2, 47) = 161.3, *P* < 0.001). Differences between groups did not reach statistical significance (Fig. 2B). The group × day interaction for IOD in CT was significant (*F*(6, 94) = 3.61, *P* = 0.003) with UnV (*P* < 0.001) and HL (*P* = 0.01) significantly reducing choroidal thinning on D8 but not on D4 (Fig. 2C). The detailed results are provided in Supplementary Table S1.

### Impact of 4 Hours of HL, UnV, and HL + UnV

For 4-hour interventions, we found a significant group × day interaction for IOD in refraction (*F*(6, 94) = 12.80, *P* < 0.001). By D4 and D8, 4 hours of HL, UnV, and HL + UnV significantly reduced myopic refraction compared to the LIM group (both for D4 and D8, *P* < 0.001). On D4 and D8, 4 hours of UnV was more effective than HL (*P* < 0.001) in reducing myopic refraction, while HL + UnV was more effective than HL in reducing myopic refraction (D4: *P* = 0.009, D8: *P* = 0.04). On D4 but not on D8, UnV was more effective than HL + UnV (*P* = 0.01) in reducing myopic refraction (Fig. 3A). A significant group × day interaction was also found for IOD in AL (*F*(6, 94) = 2.23, *P* = 0.047), with all groups showing significantly reduced axial elongation compared to that observed in the LIM group (D4: no difference between groups; D8: LIM versus UnV: *P* < 0.001; LIM versus HL: *P* = 0.01; LIM versus HL + UnV: *P* < 0.001) (Fig. 3B). Differences in AL between intervention groups did not reach statistical significance. The group × day interaction for IOD in CT was significant (*F*(6, 94) = 3.98, *P* = 0.001). On D4, differences in CT between groups did not reach statistical significance. On D8, all intervention groups prevented choroidal thinning in the myopic eye compared to the LIM group on D8 (LIM versus UnV: *P* < 0.001; LIM versus HL: *P* = 0.002; LIM versus HL + UnV: *P* = 0.002) (Fig. 3C). The detailed results are provided in Supplementary Table S1.

### Impact of 6 Hours of HL, UnV, and HL + UnV

Six-hour interventions had a significant interaction between the group and time for IOD in refraction (*F*(6, 92) = 24.67, *P* < 0.001). By D4 and D8, 6 hours of HL, UnV, and HL + UnV significantly reduced myopic refraction compared to the LIM group (both D4 and D8, *P* < 0.001). Six hours of UnV was more effective than HL in reducing myopic refraction by both D4 and D8 (*P* < 0.001). HL + UnV was more effective in reducing myopic refraction than both HL (both D4 and D8: *P* < 0.001) and UnV (only D8: *P* = 0.008) (Fig. 4A). IOD in AL had a significant group × time interaction (*F*(6, 92) = 6.4, *P* < 0.001) with all groups showing reduced axial elongation compared to the LIM eyes (LIM versus UnV: *P* < 0.001; LIM versus HL: *P* = 0.004; LIM versus HL + UnV: *P* < 0.001) on D8. On D4, only the HL + UnV group showed significantly reduced axial elongation compared with the LIM group (*P* = 0.009). HL + UnV was more effective than HL in reducing axial elongation (*P* < 0.001) on D8 but not on D4 (Fig. 4B). CT also showed a significant interaction between groups and time (*F*(6, 92) = 3.53, *P* = 0.003), with all three interventions significantly reducing choroidal thinning of the myopic eye compared to the LIM group on D8 (LIM versus all groups: *P* < 0.001) (Fig. 4C). Differences in CT between intervention groups did not reach statistical significance.

### Impact of Experimental Interventions on ACD and CCT

**Figure 5.**
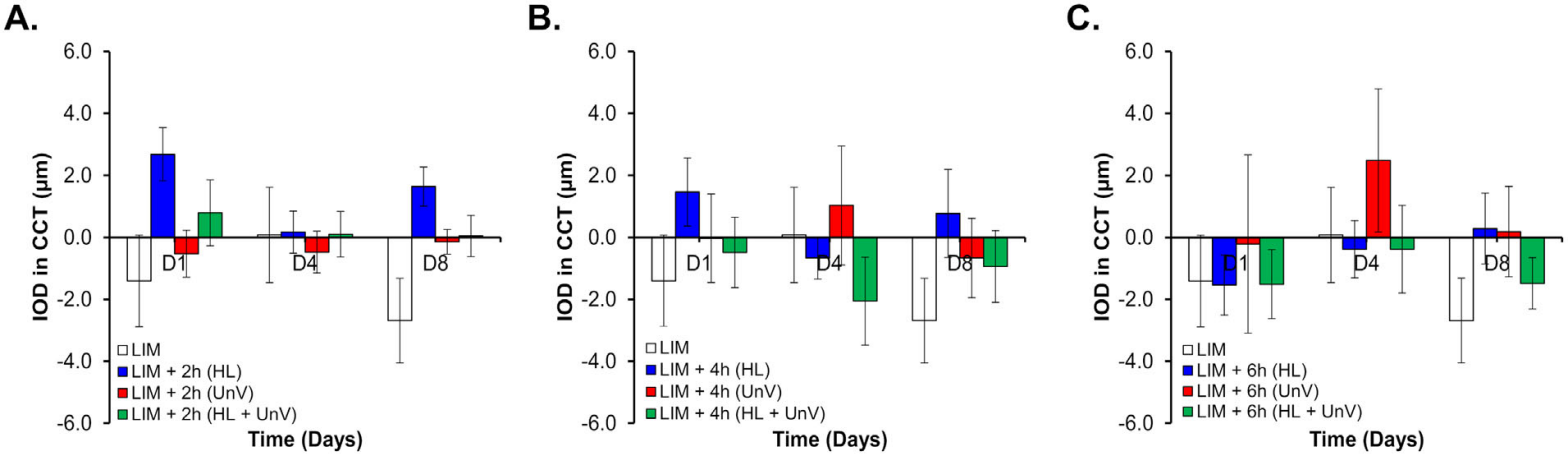
IOD in CCT on days 1, 4, and 8 of the experimental protocol in the group not exposed to any intervention (LIM) and groups exposed to 2 hours (**A**), 4 hours (**B**), and 6 hours (**C**) of HL, UnV, or both (HL + UnV).

**Figure 6.**
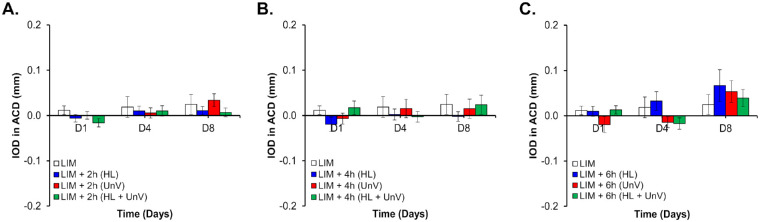
IOD in ACD on days 1, 4, and 8 of the experimental protocol in the group not exposed to any intervention (LIM) and groups exposed to 2 hours (**A**), 4 hours (**B**), and 6 hours (**C**) of HL, UnV, or both (HL + UnV).

IODs in CCT were only significantly different (*F*(3, 94) = 3.86, *P* = 0.02) between the HL and LIM groups (*P* = 0.01) for 2-hour-long interventions. IODs in ACD showed a significant effect of day only for the 2-hour (*F*(2, 94) = 3.99, *P* = 0.02) and 6-hour (*F*(2, 92) = 7.31, *P* = 0.001) interventions (Figs. 5 and 6). For more details, see Supplementary Table S1.

### Duration Response Curves on D4 and D8 of the Interventions

**Figure 7.**
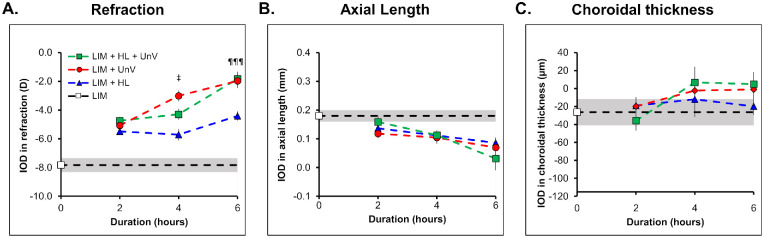
Duration-response curve for the IOD in refraction (**A**), axial length (**B**), and choroidal thickness (**C**) in the groups exposed to 2, 4, and 6 hours of HL, UnV, or both (HL + UnV) on day 4 of the experimental protocol. The LIM group that was not exposed to any intervention is represented by a *white square* and a *shaded area* for mean ± 95% confidence interval. All groups are different from each other: ^‡^*P* < 0.05. HL significantly different from both UnV and HL + UnV: ^¶¶¶^*P* < 0.001.

On D4 of the protocol, the impact of the intervention on IODs of refraction (*F*(3, 140) = 80.93, *P* < 0.001) and AL (*F*(3, 140) = 14.49, *P* < 0.001) was duration dependent. The interaction between the group and time for IOD in refraction was significant (*F*(6, 140) = 3.63, *P* = 0.002), where both 4 and 6 hours of UnV (both 4 and 6 hours: *P* < 0.001) and HL + UnV (4 hours: *P* = 0.03 and 6 hours: *P* < 0.001) were more effective than HL in reducing myopic refraction. UnV of 4 hours but not 6 hours reduced myopic refraction more than HL + UnV (*P* = 0.03) (Fig. 7A). IODs in AL and CT were not different between groups across the different durations of the interventions (Figs. 7B, 7C).

**Figure 8.**
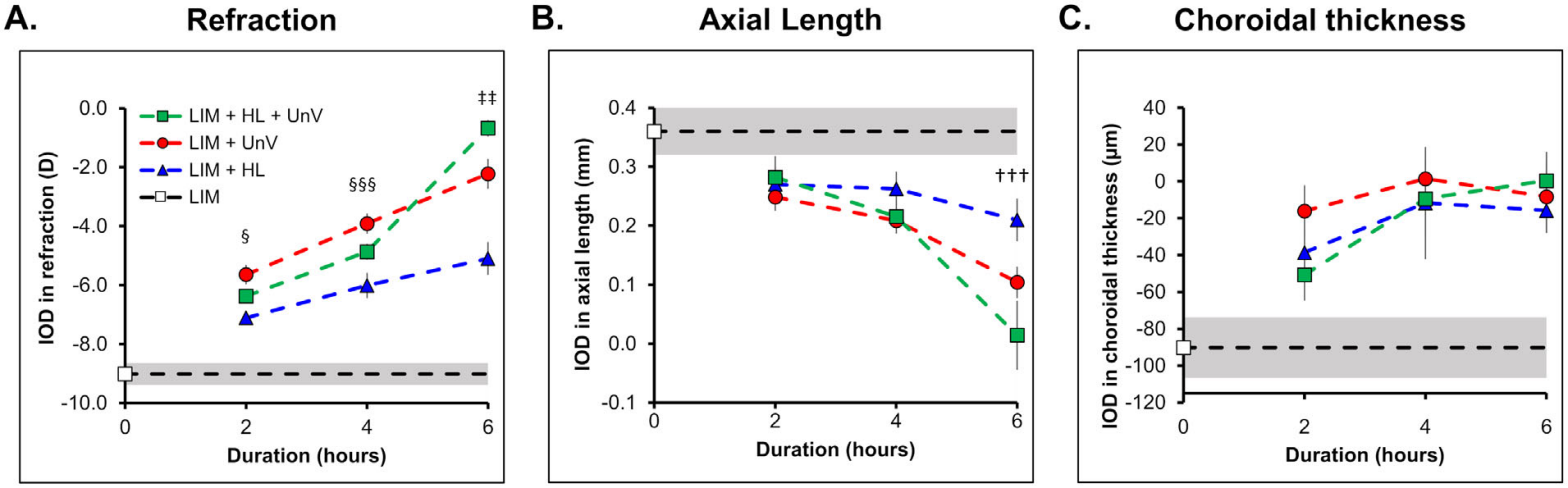
Duration-response curve for the IOD in refraction (**A**), axial length (**B**), and choroidal thickness (**C**) in the groups exposed to 2, 4, and 6 hours of HL, UnV, or both (HL + UnV) on day 8 of the experimental protocol. The LIM group that was not exposed to any intervention is represented by a *white square* and a *shaded area* for mean ± 95% confidence interval. HL is significantly different from UnV: ^§^*P* < 0.05, ^§§§^*P* < 0.001. All groups are different from each other (at least, ^‡‡^*P* < 0.01). HL is significantly different from HL + UnV (^†††^*P* < 0.001).

On D8 of the protocol, the impact of interventions (i.e., HL, UnV, HL + UnV) on IODs of refraction (*F*(2, 104) = 78.33, *P* < 0.001) and AL (*F*(2, 104) = 17.1, *P* < 0.001) was duration dependent. There was a significant interaction between the duration and type of intervention on IODs of refraction (*F*(4, 104) = 8.38, *P* < 0.001), where only 6-hour HL + UnV but not 2 or 4 hours of HL + UnV, was more effective than UnV (*P* = 0.004) and HL (*P* < 0.001) in reducing myopic refraction. Conversely, regardless of the duration, UnV was more effective than HL in reducing myopic refraction (2 hours: *P* = 0.01; 4 and 6 hours: *P* < 0.001) (Fig. 8A). Likewise, the interaction between the duration and type of intervention was significant for AL (*F*(4, 104) = 2.60, *P* = 0.04), where only 6 hours of HL + UnV was more effective than HL (*P* < 0.001) (Fig. 8B). IOD of CT was not different between intervention groups across the different durations (Fig. 8C).

## Discussion

In this study, we demonstrated that different combinations and durations of HL and UnV have varying effects on the development of LIM in a chicken model. The effect of HL alone, UnV alone, and HL + UnV in reducing myopia and AL elongation was duration dependent (IODs for refraction and AL were lowest at 6 hours, followed by 4 hours, then 2 hours). We also observed that UnV alone was more effective than HL alone in preventing LIM. Interestingly, the effect of HL + UnV in reducing LIM development was only found to be additive to a statistically significant extent (lower IODs than both UnV alone and HL alone) in the 6-hour exposure group. On D8, the greatest reduction in LIM (closest to null IODs for myopia, AL, and CT) was found in chicks subjected to 6-hour daily exposure of HL + UnV. We did not find any significant change in ACD or CCT among the groups, which was consistent with previous findings.^30^

The duration-dependent effect of UnV in reducing LIM development has been previously reported in guinea pigs,^43^ chickens,^44^ tree shrews,^33^ and marmosets.^34^ In chickens, using younger (D1–D10) and older (D7–D11) animals, Schmid and Wildsoet^44^ reported a ∼90% reduction in LIM (–10 D) by just 3 hours of normal vision or UnV per day. Contrarily, in older chicks (D7–D11) with no accommodation due to ciliary nerve section, 3 and 6 hours of UnV reduced their LIM by 60% and 96%, respectively, beyond which (i.e., 9-hour UnV) the effect plateaued. In comparison, we observed 37%, 57%, and 75% reduction in LIM by D8 on exposure to 2, 4, and 6 hours of UnV, respectively. The reduced impact of UnV that we report in our study may be due to experimental protocol differences, particularly differences in the age (visual maturation) and strain of chickens, the timing of UnV (i.e., centered at noon), experimental protocol duration, and the characteristics of the background lighting and visual-spatial environments during normal vision/UnV. In addition, Nickla et al.^45^ showed less ocular growth and LIM during the afternoon than in the morning in response to –10 D defocus in chicks. The authors also found UnV to be more effective to reduce LIM in the evening than in the morning.^46^ From our findings, it can be argued that, when centered at noon, longer durations of continuous UnV, spilling further into the afternoon (i.e., 2 hours: 11 am to 1 pm; 4 hours: 10 am to 2 pm; 6 hours: 9 am to 3 pm), would potentiate the impact of UnV further. Conversely, here we show a newfound duration-dependent impact of HL on reducing myopic shift and axial elongation in LIM. There is currently insufficient evidence linking the total quantum of light exposure (intensity of the exposure × daily duration of the exposure × duration of the study protocol) with LIM reduction, but a recent study investigating LIM in mice showed that retinal expression of DA-related genes and proteins increased with increasing intensities of light,^38^ suggesting a dose-dependent release of ocular growth neuromodulators like DOPAC, a metabolite of DA, and/or nitric oxide (NO).^38^^,^^47^^,^^48^ Whether this release is dependent on the total quantum of light, whereby the retina acts as a photon counter to drive neuromodulatory responses, remains unclear yet unlikely, given that short intermittent light pulses have been reported to be more effective than equiluminant continuous light in chickens.^49^ In humans, myopia progression rates in children are slower during a 6-month period that included summer vacation, when days are longer and brighter, compared to winter. These findings partially support an association between the total quantum of light exposure and myopia control.^50^ On the other hand, it is worth mentioning that Stone and colleagues^51^ reported only a partial and transient effect (i.e., on D4 but not on D11 of the authors’ experimental protocol) of natural high-intensity daylight on FDM in chicks reared outdoors, without any established associations with the retinal DA levels. Given that our experimental protocol was only 8 days long, a longer investigation with more frequent data sampling is required to confirm the permanency of the duration-dependent impact of HL on LIM.

Next, UnV alone was more effective than HL alone in reducing LIM development. During UnV, a temporary myopic defocus occurs, while in HL, the hyperopic defocus is constantly present in eyes with LIM. Studies had shown that when the eye receives competing defocus signals, the more myopic defocus dominates ocular growth control^52^^–^^54^; this could explain the stronger emmetropization signal from UnV. Another suggestion for the disparity involves UnV being a visually/optically guided feedback process,^15^^,^^52^ with an endpoint of refractive error elimination. In contrast, HL acting through a potentially distinct pathway involves photoreceptor stimulation and retinal neuromodulation^26^^,^^29^^,^^30^ that can only marginally overcome the existing visual experience (i.e., hyperopic defocus). Whether UnV remains a more potent driver for emmetropization if the myopiagenic stimulus is reduced (e.g., using a –5 D defocus instead of –10 D) remains to be investigated. Additionally, axial myopic retinas are more prolate (flatter in periphery) and have a relative peripheral hyperopia compared to hyperopic eyes with oblate retinas (steeper in periphery) and relative peripheral myopia.^55^^,^^56^ Relative peripheral refraction (hyperopia or myopia) compared to the central refraction may exacerbate or reduce myopia development, respectively,^55^^,^^56^ and can partially explain a greater drive of UnV alone in reducing LIM compared to HL alone.

Our data did not demonstrate that the combined effect of HL + UnV in reducing LIM development was additive, when interventions were 2 or 4 hours long. These findings suggested that UnV and HL may be deploying different pathways of compensatory mechanisms in LIM.^18^ In chicks, Ashby et al.^29^ found that on day 5, the protective effects of diffuser removal against FDM was significantly enhanced by 15 minutes of high-intensity indoor lights (15,000 lux) during diffuser removal. This was not in agreement with our findings, where at both D4 and D8, the protective impact of HL + UnV on AL increase, myopic refraction, and choroidal thinning was similar if not worse than UnV alone for 2- and 4-hour-long exposures. The additional alleviation from FDM observed by Ashby and colleagues may result from higher exposure levels to HL and DA synthesis due to diffuser removal,^36^^–^^38^ while in LIM, there was only a minimal increase in HL levels reaching the eye associated with UnV. In addition, Ashby et al.^29^ reported that the spectral distribution of their experimental halogen lights (300–1000 nm, peaking at 700 nm) was similar to daylight over the range of visible light for chickens (360–700 nm),^57^ while our experimental light had a typical LED spectrum peaking around 449 nm and 583 nm (Fig. 1). The fullness of the halogen light spectrum light may have promoted the impact of short-duration, high-intensity light on the recovery from FDM.^28^ Parenthetically, studies investigating the impact of lens or diffuser removal should take into account the different spatial characteristics of the housing environment.^12^^,^^39^ Another plausible explanation for our findings was that the significant increase in pupillary constriction induced by HL, during UnV, increased the depth of focus and attenuated the accuracy of the UnV drive. Pupillary constriction under HL may also explain the reduced impact of defocus on LIM but failed to explain the potentiating effect of HL on lens-induced hyperopia.^30^

Interestingly, HL and UnV were additive to a statistically significant extent when interventions were 6 hours long and almost completely compensated for LIM (IOD: –0.68 D), AL (IOD: 0.01 mm), and CT (IOD: 0.46 µm) by D8 (Supplementary Table S1). These findings suggested a duration-dependent synergy/intermodulation between HL and UnV. While we do not have an understanding of the mechanisms to explain these data yet, this duration-dependent synergy may originate from delayed photobiomodulatory processes originating at the retinal level that enhance the drive for emmetropization conferred by UnV.^58^ Vitreal DOPAC (a metabolite of DA) levels are known to increase with both the duration^59^ and intensity^37^ of light exposure in chickens, with the circadian rhythm of DA release observed to peak at 12 hours into the light phase.^59^ DA can trigger the release of other neurotransmitters like NO,^47^ reducing LIM either alone or in conjunction. This DA release is shown to depend on not only the retinal luminance but also the image contrast/visual spatial information,^60^ which gets altered during normal vision or UnV. It is possible that the combination of UnV and HL for 6 hours surpassed a retinal DA threshold that enhanced recovery from LIM further, through an overlapping pathway between UnV and HL. It is worth highlighting that the trend of HL and UnV alone was unaltered throughout the different durations of exposure.

In existing literature, chicks had shown changes in CT in response to altered retinal image quality, and it had been suggested that this CT is a part of mechanisms regulating ocular growth and emmetropization.^2^^,^^61^^,^^62^ As expected, in our study, LIM led to choroidal thinning.^45^^,^^46^ While eyes exposed to 4 and 6 hours of HL, UnV, or HL + UnV all had significantly thicker choroids compared to LIM eyes, 2 hours of HL and UnV but not HL + UnV led to significantly thicker choroids than LIM eyes, corroborating the observed nonadditivity of 2-hour-long HL and UnV exposures for AL and refraction. A reduction in choroidal thinning was particularly observed when the duration of interventions was increased from 2 to 4 hours. Although these findings may be explained by a rise in intraocular temperature, leading to increased permeability and vasodilation of choroidal vessels, and a release of neurotransmitters resulting in choroidal thickening,^61^^,^^62^ it is unlikely, yet untested, that our wide-field HL intervention caused any changes in intraocular temperature. Nonetheless, the synergy between HL and UnV at 6-hour interventions seems to be independent of choroidal thickening induced by HL as 4 hours of HL yielded similar choroidal thickening as 6 hours of HL.

Our study has a few limitations. First, we have opted for an environment lacking color, movement, and other usual features of a visual environment for the housing of the chicks. This environment lacks fine spatial details and may not be the most suitable environment for emmetropization or the recovery from LIM.^12^ Second, given the differences in ocular optics, anatomy, and physiology between chickens and humans,^63^ it was difficult to directly contextualize and translate our findings to humans. While our work is in agreement with the literature that removing hyperopic defocus reduces myopia development, such findings are applicable to animal models with normally functioning emmetropization yet potentially less so to myopic human subjects with deficiencies in the emmetropization mechanism.^64^ Finally, our findings suggest that exposure to HL can slow the development of myopia in a duration-dependent manner regardless of the refractive status of the eye or concurrent myopiagenic stimuli (e.g., near visual work). In addition, the combination of high-intensity light exposure and visual breaks (e.g., reducing accommodative lag) may have the highest potential to prevent myopia development, when applied for long durations during daytime (50% of daytime here). These lengthy interventions may not be implementable in real life.

## Conclusion

In this study, we showed that daily exposure to 2, 4, or 6 hours of HL or UnV slows the shift to myopic refraction in chickens in a duration-dependent manner. Combined with UnV, only 6 hours of HL fully halted the development of LIM (i.e., axial elongation, myopic refraction, and choroidal thinning). The synergetic effect of HL and UnV is dependent on the duration of the intervention. Further molecular work is required to better understand this peculiar synergy between HL and UnV, with the potential of translating such findings into pharmacologic interventions or combined light/optics interventions.

## Supplementary Material

Supplement 1

## Appendix

Amended April 4, 2023: Reference 41 cited a meeting abstract, although the work had been published after the meeting in a full article. Reference 41 has now been updated from “Najjar R, Chao De La, Barca JM, Barathi VA, et al. Ocular growth and metabolomics are dependent upon the spectral content of white light. *Invest Ophthalmol Vis Sci.* 2018;59(9):755” to “Najjar RP, Chao De La Barca JM, Barathi VA, et al. Ocular growth and metabolomics are dependent upon the spectral content of ambient white light. *Sci Rep.* 2021;11(1):7586.”
