# Supplementary material for: A Duration-Dependent Interaction Between High-Intensity Light and Unrestricted Vision in the Drive for Myopia Control
Source: Invest Ophthalmol Vis Sci. 2023 Mar 23;64(3):31. doi: 10.1167/iovs.64.3.31 (PMC10050902; doi:10.1167/iovs.64.3.31)
Supplement: Supplement 1 [file iovs-64-3-31_s001.pdf]

Supplementary Material

A duration-dependent interaction between high-intensity light and unrestricted vision in the drive for myopia control

Sayantan Biswas[1], Arumugam R. Muralidharan[1,2], Bjorn Kaijun Betzler[3], Joanna Marie Fianza Busoy[1], Veluchamy A. Barathi[1,2,3], Royston K. Y. Tan[1,2], Wan Yu Shermaine Low[1], Dan Milea[1,2,4], Biten K. Kathrani[5], Noel A. Brennan[5], Raymond P. Najjar[1,2,6,7*]

Supplementary Table

Supplementary table 1: Changes in ocular measurements in groups exposed to 0h, 2h, 4h and 6h of high-intensity light, optical refocus or both. Data represented as mean ± SEM of the inter-ocular difference between (i.e., experimental eye - control eye).

Ocular parameter	Duration of the intervention (hours)	Intervention	Days			P-values 2W RM ANOVA		
			D1	D4	D8	Group	Day	Group × Day
Refraction (D)	0	LIM	-0.05 ± 0.28	-7.83 ± 0.48	-9.02 ± 0.37	-		
Axial length (mm)			0.00 ± 0.02	0.18 ± 0.02	0.36 ± 0.04			
Choroidal thickness (µm)			15.04 ± 10.35	-26.35 ± 14.55	-90.27 ± 16.44			
ACD (mm)			0.01 ± 0.01	0.02 ± 0.02	0.02 ± 0.02			
CCT (µm)			-1.41 ± 1.48	0.08 ± 1.54	-2.69 ± 1.37			
Refraction (D)	2	LIM + HL	-0.08 ± 0.20	-5.49 ± 0.18	-7.11 ± 0.20	<0.001	<0.001	<0.001
		LIM + UnV	-0.27 ± 0.24	-5.08 ± 0.36	-5.65 ± 0.33			
		LIM + HL + UnV	0.16 ± 0.19	-4.75 ± 0.28	-6.37 ± 0.22			
Axial length (mm)		LIM + HL	0.01 ± 0.01	0.14 ± 0.02	0.27 ± 0.03	0.092	<0.001	0.196
		LIM + UnV	0.01 ± 0.02	0.12 ± 0.01	0.25 ± 0.02			
		LIM + HL + UnV	0.01 ± 0.02	0.16 ± 0.02	0.28 ± 0.04			
Choroidal thickness (µm)		LIM + HL	-6.08 ± 6.14	-19.04 ± 9.65	-38.65 ± 13.39	0.206	<0.001	0.003
		LIM + UnV	7.73 ± 5.87	-33.62 ± 8.88	-16.12 ± 13.94			
		LIM + HL + UnV	-9.00 ± 12.33	-35.83 ± 11.04	-50.67 ± 13.92			
ACD (mm)		LIM + HL	-0.01 ± 0.01	0.01 ± 0.01	0.01 ± 0.01	0.576	0.022	0.845
		LIM + UnV	0.00 ± 0.01	0.01 ± 0.01	0.03 ± 0.01			
		LIM + HL + UnV	-0.02 ± 0.01	0.01 ± 0.01	0.01 ± 0.01			

CCT (µm)		LIM + HL	2.68 ± 0.86	0.17 ± 0.68	1.64 ± 0.63	0.015	0.59	0.274
		LIM + UnV	-0.53 ± 0.76	-0.48 ± 0.67	-0.15 ± 0.40			
		LIM + HL + UnV	0.79 ± 1.07	0.10 ± 0.74	0.04 ± 0.66			
Refraction (D)	4	LIM + HL	0.00 ± 0.14	-5.71 ± 0.41	-6.01 ± 0.43	<0.001	<0.001	<0.001
		LIM + UnV	0.01 ± 0.18	-3.01 ± 0.40	-3.92 ± 0.35			
		LIM + HL + UnV	-0.05 ± 0.38	-4.30 ± 0.38	-4.87 ± 0.26			
Axial length (mm)		LIM + HL	0.01 ± 0.02	0.11 ± 0.02	0.26 ± 0.03	0.001	<0.001	0.047
		LIM + UnV	-0.04 ± 0.02	0.10 ± 0.02	0.21 ± 0.02			
		LIM + HL + UnV	-0.01 ± 0.01	0.11 ± 0.01	0.22 ± 0.03			
Choroidal thickness (µm)		LIM + HL	-3.42 ± 6.85	-12.08 ± 19.77	-11.73 ± 30.42	0.062	0.066	0.001
		LIM + UnV	-15.58 ± 8.95	-2.33 ± 9.97	1.46 ± 14.93			
		LIM + HL + UnV	-1.92 ± 12.80	6.77 ± 17.47	-9.38 ± 20.20			
ACD (mm)		LIM + HL	-0.02 ± 0.02	0.00 ± 0.01	0.00 ± 0.01	0.507	0.42	0.822
		LIM + UnV	-0.01 ± 0.01	0.02 ± 0.02	0.02 ± 0.02			
		LIM + HL + UnV	0.02 ± 0.01	0.00 ± 0.01	0.02 ± 0.02			
CCT (µm)		LIM + HL	1.46 ± 1.11	-0.67 ± 0.68	0.77 ± 1.42	0.332	0.696	0.572
		LIM + UnV	-0.03 ± 1.43	1.03 ± 1.92	-0.67 ± 1.28			
		LIM + HL + UnV	-0.49 ± 1.14	-2.06 ± 1.42	-0.94 ± 1.16			
Refraction (D)	6	LIM + HL	0.12 ± 0.28	-4.41 ± 0.31	-5.10 ± 0.56	<0.001	<0.001	<0.001
		LIM + UnV	0.00 ± 0.32	-1.97 ± 0.55	-2.23 ± 0.51			
		LIM + HL + UnV	-0.09 ± 0.23	-1.83 ± 0.49	-0.68 ± 0.28			
Axial length (mm)		LIM + HL	0.01 ± 0.02	0.09 ± 0.02	0.21 ± 0.04	<0.001	<0.001	<0.001
		LIM + UnV	-0.01 ± 0.03	0.07 ± 0.03	0.10 ± 0.03			
		LIM + HL + UnV	0.02 ± 0.03	0.03 ± 0.04	0.01 ± 0.06			
Choroidal thickness (µm)		LIM + HL	-3.23 ± 12.24	-19.85 ± 9.53	-15.85 ± 12.24	0.004	0.003	0.003
		LIM + UnV	6.91 ± 7.45	-1.05 ± 15.86	-8.32 ± 18.42			
		LIM + HL + UnV	1.49 ± 6.97	4.87 ± 13.47	0.46 ± 15.57			
ACD (mm)		LIM + HL	0.01 ± 0.01	0.03 ± 0.02	0.07 ± 0.3	0.382	0.001	0.433
		LIM + UnV	-0.02 ± 0.02	-0.01 ± 0.01	0.05 ± 0.02			
		LIM + HL + UnV	0.01 ± 0.01	-0.02 ± 0.01	0.04 ± 0.02			
CCT (µm)		LIM + HL	-1.54 ± 0.97	-0.38 ± 0.93	0.28 ± 1.14	0.437	0.21	0.876
		LIM + UnV	-0.21 ± 2.88	2.48 ± 2.31	0.18 ± 1.86			
		LIM + HL + UnV	-1.51 ± 1.11	-0.38 ± 1.41	-1.49 ± 0.83			

All values are expressed as the mean inter-ocular difference between experimental and control eyes ± SEM. 2W RM ANOVA: Two-way repeated measures analysis of variance, LIM: Lens induced myopia, HL: High intensity light, UnV: Unrestricted vision, ACD: Anterior chamber depth, CCT: Central corneal thickness
